# RISING beamline (BL28XU) for rechargeable battery analysis

**DOI:** 10.1107/S1600577513025733

**Published:** 2013-11-28

**Authors:** H. Tanida, K. Fukuda, H. Murayama, Y. Orikasa, H. Arai, Y. Uchimoto, E. Matsubara, T. Uruga, K. Takeshita, S. Takahashi, M. Sano, H. Aoyagi, A. Watanabe, N. Nariyama, H. Ohashi, H. Yumoto, T. Koyama, Y. Senba, T. Takeuchi, Y. Furukawa, T. Ohata, T. Matsushita, Y. Ishizawa, T. Kudo, H. Kimura, H. Yamazaki, T. Tanaka, T. Bizen, T. Seike, S. Goto, H. Ohno, M. Takata, H. Kitamura, T. Ishikawa, T. Ohta, Z. Ogumi

**Affiliations:** a Kyoto University, Japan; b JASRI/SPring-8, Japan; c Riken Harima Institute, Japan; d Ritsumeikan University, Japan

**Keywords:** lithium-ion battery, *in situ* analysis, time X-ray diffraction, X-ray absorption fine structure

## Abstract

The newly installed BL28XU beamline at SPring-8 is dedicated to *in situ* structural and electronic analysis of rechargeable batteries. It supports the time range (1 ms to 100 s) and spatial range (1 µm to 1 mm) needed for battery analysis. Electrochemical apparatus for battery charging and discharging are available in experimental hutches and in a preparation room. Battery analysis can be carried out efficiently and effectively using X-ray diffraction, X-ray absorption fine-structure analysis and hard X-ray photoelectron spectroscopy. Here, the design and performance of the beamline are described, and preliminary results are presented.

## Abstract 2

The BL28XU beamline, dedicated to rechargeable battery analysis, is described.

## 1. Introduction

**Figure 1:**
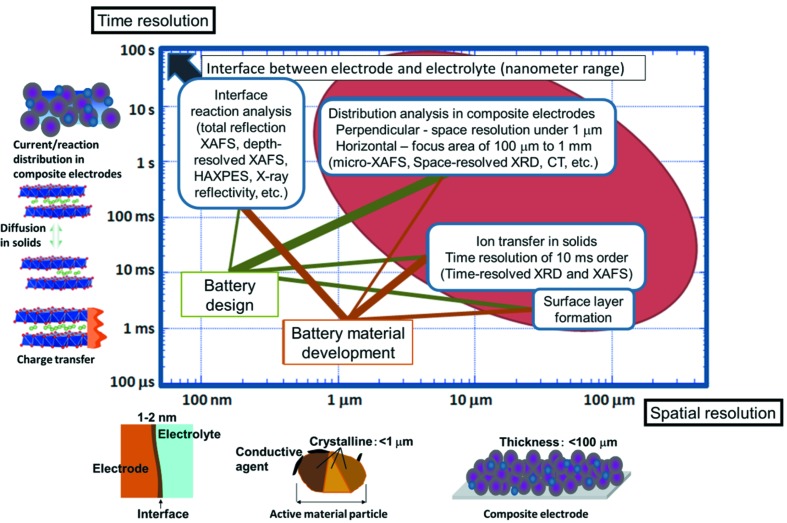
Time and spatial resolution required for battery reactions.

The lithium-ion rechargeable battery (LIB) is a key component technology for electric vehicles and smart electrical grids. Understanding the reactions inside a LIB is indispensable for elucidating and improving battery properties such as the accumulated energy, power, durability and safety. However, the dynamics of the battery reactions remain unclear because LIBs are generally tightly sealed, making it difficult to investigate the phenomena inside. The reactions in the electrodes, electrolyte solutions and electrode/electrolyte interfaces take place at various times and in various spatial ranges as shown in Fig. 1 ▶. A typical LIB consists of a cathode made of a transition metal oxide, an anode made of graphite, a separator diaphragm and electrolyte solutions. Lithium ions migrate from the cathode and are intercalated into the anode during charge, and migrate to the cathode during discharge. Practical LIBs have highly heterogeneous composite electrodes composed of powders of active materials mixed with a small amount of carbon powder as conductive agents and binder. The size of the reaction interface, the size of the active material particles, and the thickness of the composite electrodes range from 1 nm to 0.1 mm. Charge transfer in electrodes, lithium ion migration and structural relaxation for phase transitions take from 0.1 ms to 100 s. Because of the distinctive structure of the composite electrodes, the total battery reaction comprises various elementary reaction processes with various time and spatial ranges. These reaction phenomena during battery charge and discharge should be completely clarified using advanced time-resolved and spatially resolved experimental techniques.

Synchrotron radiation is useful for observing phenomena inside batteries due to its high penetration property and wide time and spatial resolutions. Powerful methods for clarifying reactions inside batteries include X-ray absorption fine structure (XAFS) (Nakai *et al.*, 1997 ▶; Mansour *et al.*, 1999 ▶; Kim & Cho, 2009 ▶; Dominko *et al.*, 2009 ▶; Chen *et al.*, 2009 ▶) for the electronic structure of the transition metal in the cathode material, X-ray diffraction (XRD) (Yang *et al.*, 2000 ▶; Balasubramanian *et al.*, 2001 ▶; McBreen, 2009 ▶; Orikasa *et al.*, 2013 ▶) for the crystal structure of the cathode active material, and hard X-ray photoelectron spectroscopy (HAXPES) (Edström *et al.*, 2004 ▶; Shikano *et al.*, 2007 ▶; Takanashi *et al.*, 2011 ▶) for the electronic structure of the electrode surface. Reactions at the interface between the electrolyte and an electrode were recently investigated under *in situ* working conditions by using *in operando* experiments (Leriche *et al.*, 2010 ▶) and total-reflection X-ray absorption spectroscopy (XAS) (Takamatsu *et al.*, 2012 ▶).

The analysis of battery reaction phenomena requires special experimental tools such as an environmental control unit and charge–discharge equipment. These tools along with the X-ray methods mentioned above are needed to elucidate the dynamics of the battery reactions. A glove box and environmental tester with a thermostat dedicated to battery sample are also desirable for preparing and testing samples in a preparation room.

The new beamline, BL28XU, at SPring-8 is dedicated to *in situ* structural and electronic analysis of rechargeable batteries using X-ray analyses and these essential tools for investigating battery reactions. SPring-8, the largest third-generation synchrotron radiation facility, provides an exceptionally high brilliance and high-energy photon beam for time-resolved and spatially resolved experiments. The energy range from 5 to 30 keV covers the absorption edges of Mn, Fe, Co and Ni, which are commonly used active materials for the cathodes of LIBs. The high penetration property enables *in situ* measurement without having to disassemble batteries. The wide time and spatial resolutions facilitate analysis and elucidation of battery reactions, which should lead to battery designs and materials needed for next-generation batteries (Matsubara, 2012 ▶).

The construction of the beamline was directed by the Research and Development Initiative for Scientific Innovation of New Generation Batteries (RISING) project funded by Japan’s New Energy and Industrial Technology Development Organization (NEDO) and with technical support from the Japan Synchrotron Radiation Research Institute (JASRI) and the RIKEN Harima Institute. The RISING project, which started in 2009, is an all-Japan project with robust industry–government–academia collaboration. Its goal is to develop innovative rechargeable batteries with an energy density of initially 300 W h kg^−1^ and ultimately 500 W h kg^−1^ as well as to further improve LIB performance through strong cooperation among universities, industries and national institutes. Here, the design and performance of the beamline are described, and preliminary results are presented.

## 2. Beamline overview

**Figure 2:**
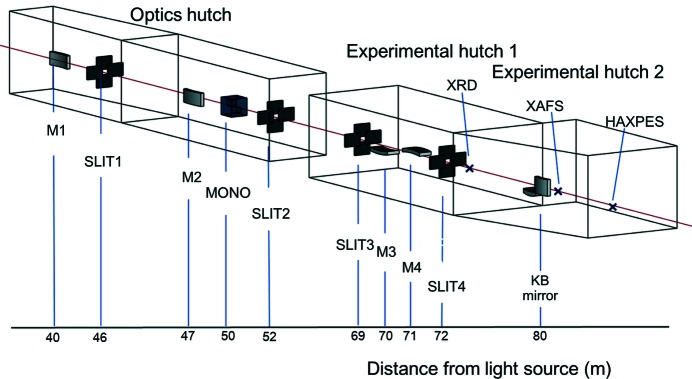
Schematic of beamline BL28XU and distances from the light source. Three hutches: optics, experimental 1, experimental 2. Main components: first mirror (M1), second mirror (M2), channel-cut compact monochromator (MONO), four-jaw slits (SLIT1-4), third mirror (M3), fourth mirror (M4). Sample positions are at XRD, XAFS and HAXPES.

The BL28XU beamline uses a SPring-8 standard synchrotron light source and optics for quick-scanning XAFS measurement (Hirose, 2010 ▶; Sekizawa *et al.*, 2013 ▶). Quasi-monochromatic X-rays are obtained using an in-vacuum tapered undulator to extend the energy bandwidth to 2 keV. As shown in Fig. 2 ▶, there are three hutches: one optics and two experimental. The experimental hutches are located 75–80 m from the light source to increase the reduction ratio of the focusing mirror.

There are four Si-coated mirrors with Rh and Pt stripes. The first mirror (M1) is a liquid-nitrogen-cooled horizontal-deflection flat mirror that reduces the heat load from the light source. The second mirror (M2) is a water-cooled spherically bent mirror that horizontally focuses the X-ray beam towards one of the experimental hutches to 200 µm. These two mirrors are 1 m-long and have a glancing angle of 2 mrad. They are positioned so as to separate the horizontally reflected X-ray beam from the higher-energy X-rays emitted from the light source. The third and fourth mirrors (M3 and M4) are water-cooled spherically bent mirrors that vertically focus the X-ray beam to 100 µm and reject the higher harmonics. They are 700 mm-long. The use of these mirrors enables focused and very low divergence beams to be obtained at the sample.

A servomotor-driven compact monochromator (MONO) with a Si 111 channel-cut crystal (Nonaka *et al.*, 2012 ▶) located downstream of M2 and cooled with liquid nitrogen is used to adjust the heat load of the monochromatic beam (5–30 keV). The high brilliance of the X-ray beam enables the channel-cut crystal to have a very narrow gap (3 mm). The photon flux is around 1 × 10^14^ photons s^−1^. The vertical deflection of the beam from the monochromator and the vertical focusing mirrors is very small, and the beam position at the focal point is virtually constant, which is advantageous for measuring the reaction distribution and grazing incidence of battery samples. A Kirkpatrick–Baez (KB) mirror system (JTEC, Japan) is available for use with the 1 µm focused X-ray beam. The available beam sizes are 200 µm (H) × 100 µm (V) with M3 and M4, and 1.0 µm × 1.0 µm with the KB mirror system. The main beamline parameters are summarized in Table 1 ▶.

## 3. Ancillary facilities

Experimental hutch 1 is used for conducting time-resolved XRD experiments during battery reactions with a time resolution of 100 ms. An anomalous X-ray scattering method along with a narrow-gap channel-cut monochromator and an eight-circle diffractometer is used for element- and valence-selective diffraction measurements. A grazing-incidence XRD and X-ray reflection method are used to analyze the structural changes in the active material and at the electrolyte interface during the reaction.

Experimental hutch 2 is used for conducting spectroscopic experiments using time-resolved and spatially resolved XAFS measurements and HAXPES measurements. It supports time-resolved XAFS measurements using the quick-scanning XAFS measurements (Frahm, 1988 ▶) with a servomotor-driven channel-cut monochromator, and spatially resolved XAFS measurements with the beam microfocused using the KB mirror system. Combining the diffraction and spectroscopic measurements with various measurement times and probe sizes enables the investigation of complex phenomena in both model and commercial batteries.

## 4. Highlights

### 4.1. Chemical mapping of LiCoO_2_ particles using microfocused beam

The thickness of a composite LIB electrode is typically 10–200 µm (Johnson & White, 1998 ▶). The size of an active material particle in the electrode is 10 nm to 10 µm depending on the materials and preparation conditions. The electrode reaction is inhomogeneously distributed in the electrode (Liu *et al.*, 2010 ▶). However, the distribution of the reactivity has not been clearly understood, especially within single particles of electrode materials. A microbeam X-ray mapping method is useful for making fine observations of single particles in composite electrodes down to micrometer resolution.

A model electrode was prepared by spreading LiCO_2_ particles in a single layer on an LIB electrode. The LiCO_2_ particles, carbon black and polyvinylidene fluoride (Kureha Chemical Industry) were mixed at a weight ratio of 5:50:45 with 1-methyl-2-pyrrolidone (Wako Chemicals, 99%). The slurry was coated onto an aluminium foil current collector and dried in a vacuum oven at 353 K. The particles had a size of 2–15 µm, as determined from scanning electron microscopy measurement. They were thus similar in size to previously reported ones (Sheu *et al.*, 1997 ▶; Nakamura & Kajiyama, 1999 ▶; Wang *et al.*, 1999 ▶; Lu & Yeh, 2001 ▶).

Microbeam X-ray mapping spectra were measured by focusing the X-ray beam onto the sample using the Rh-coated KB mirror system with a 5 mrad glancing angle. The spatial resolution was 1.0 µm × 1.0 µm, and the photon flux was 10^11^ photons s^−1^. Transmission measurements were made using two ionization chambers. A chemical mapping image was measured using a scanning method with a two-axis stepper-motor translation stage. XAFS spectra were measured at 5 s per spectrum with a quick-scanning method for the initial state of the battery reaction of LiCoO_2_ single particles.

**Figure 3:**
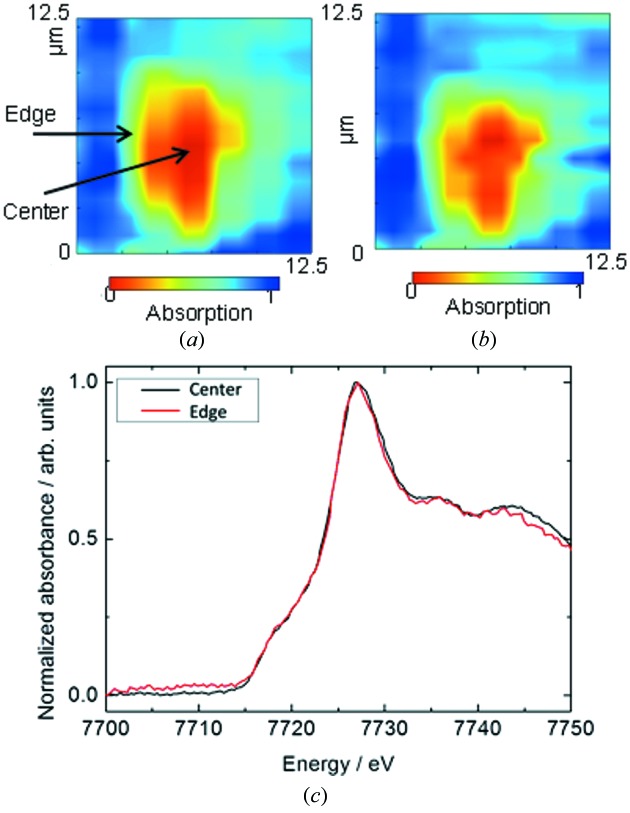
X-ray transmission images of a LiCoO_2_ particle obtained using a 1 µm beam at photon energies of (*a*) 7.71 keV and (*b*) 7.8 keV. (*c*) Co *K*-edge XAFS spectra at the centre and edge of a particle.

Two-dimensional X-ray transmission images of a LiCoO_2_ particle were measured by scanning the sample at 7.71 and 7.8 keV, which are beyond and above the Co *K*-edge absorption energy, respectively. The X-ray beam position must be fixed during the energy scan of the XAFS measurement. As shown in Figs. 3(*a*) and 3(*b*) ▶, the displacement of the X-ray beam from the KB mirror between the two energy points was negligible compared with the particle size. This means that this beamline supports X-ray mapping with 1 µm resolution.

Analysis of battery reactions requires not only spatial resolution but also time resolution because the state of the electrode materials continuously changes during a charge–discharge reaction in accordance with the extraction–insertion reaction of lithium ions. Images for the initial state of the battery reaction were obtained by acquiring the detector signals on-the-fly while the sample stage was moved for a few minutes. The spectra at the centre and edge points of a single LiCoO_2_ particle were almost the same as shown in Fig. 3(*c*) ▶. This is because a reaction has not yet distributed at the initial state of the battery reaction. This method can thus be applied to *in situ* time-resolved and spatially resolved measurement for reaction analysis of a single particle in the electrode under battery cell use conditions.

Furthermore, if a reaction map for a higher rate and a wider area is needed, a two-dimensional detector can be used for the full-field imaging (Haibel *et al.*, 2010 ▶; Meirer *et al.*, 2011 ▶; Tanida *et al.*, 2011 ▶, 2013 ▶). For a dilute sample, fluorescence X-rays emitted from the sample are detected using a 21-element pure Ge pixel array detector (Canberra, USA). A wide variety of analyses of the reaction distribution in LIBs can be performed by using the microfocusing KB mirror system, a fixed beam position at the focal point, and a high-rate energy scan of the monochromator.

### 4.2. *In situ* time-resolved XRD for LiNi_0.5_Mn_1.5_O_4_

The charge–discharge reaction in LIBs causes the crystal structure of the active materials to change with the electric potential (Orikasa *et al.*, 2013 ▶). *In situ* time-resolved XRD is a powerful technique for analyzing the phase change of active materials during non-equilibrium battery reaction without having to disassemble the battery. The model material used was LiNi_0.5_Mn_1.5_O_4_, which has three phases in the charge–discharge reaction, LiNi_0.5_Mn_1.5_O_4_ (Li1), Li_1/2_Ni_0.5_Mn_1.5_O_4_ (Li1/2) and Ni_0.5_Mn_1.5_O_4_ (Li0). In an equilibrium state there are two phase coexistence regions (Ariyoshi *et al.*, 2004 ▶; Kim *et al.*, 2004 ▶).

Electrode cells packed with laminated Al films were prepared. The reference and counter electrodes were Li foil. During a 1 h charge reaction (1 C rate), XRD patterns were measured by using a pixel array detector, PILATUS 100K (DECTRIS, Switzerland), at an exposure time of 500 ms. The experimental set-up is reported elsewhere (Arai *et al.*, 2013 ▶).

**Figure 4:**
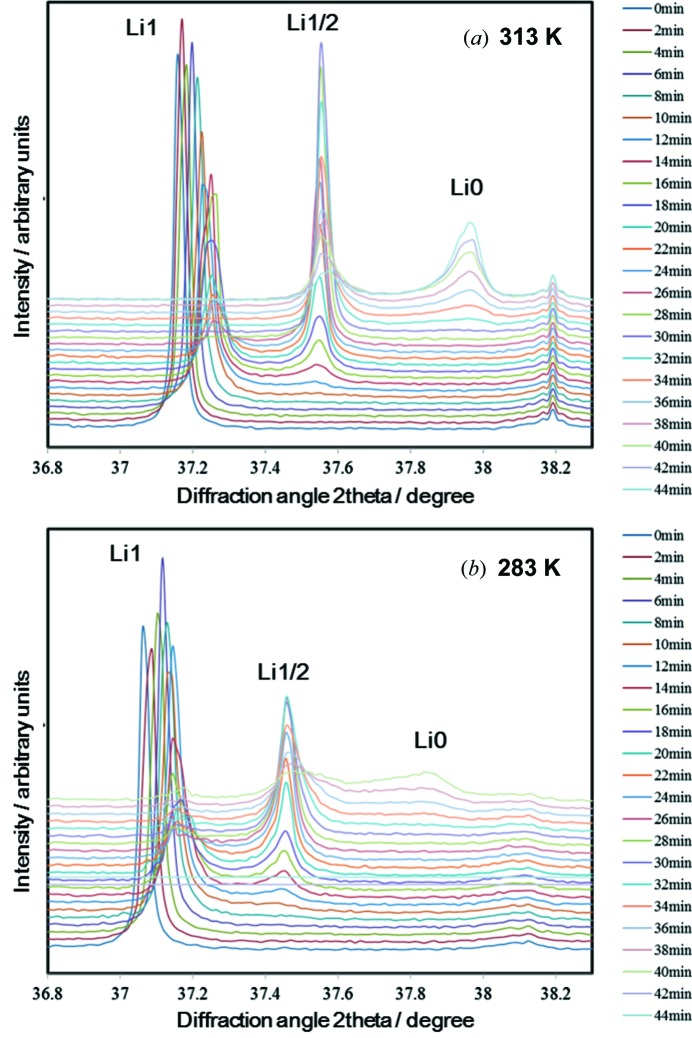
Time-resolved XRD patterns for Li_1–*x*_Mn_0.5_Ni_1.5_O_4_ during charge cycle for 1 h at (*a*) 313 K and (*b*) 283 K.

Time-resolved XRD patterns of (115) diffraction for LiNi_0.5_Mn_1.5_O_4_ were obtained by using 1 Å X-ray wavelength during charge reaction cycles of 1 h at 313 K and 283 K. The upper voltage was limited to 4.8 V. In the 313 K measurement the charge reaction was stopped at 40 min. As shown in Figs. 4(*a*) and 4(*b*) ▶, three peaks corresponding to the Li1, Li1/2 and Li0 phases were obtained during the charging reaction cycle. The peak shift of Li1 towards a higher angle during the initial stage of charging is ascribed to the solid solution region near the Li1 phase, which corresponds to the contraction of the Li1 lattice. The peak shifts were observed not to depend on the temperature, and a phase transition from the Li1 phase to the Li1/2 phases appeared at both temperatures. However, the appearance of the peak from Li0 depended on the temperature. Comparison of the Li0 peak intensities at 313 K and 283 K shows that the formation of the Li0 phase at 283 K trailed that at 313 K for the same charge current. This indicates that the phase transition reaction is much slower than the lithium-ion extraction reaction.

The time resolution depends on the detector. A scintillation counter with an eight-circle diffractometer is used for precision analysis of the crystal structure. One- or two-dimensional detectors are used for time-resolved measurement by simultaneously obtaining a few diffraction peaks. This enables analysis of the diffusion or phase transition of the material structure. The high-brilliance and high-energy X-rays (5–30 keV) of this beamline make it useful for a wide variety of samples, from powder, film and electrodes to commercial batteries.

## 5. Conclusion

Several RISING project experiments using BL28XU were conducted in April 2012. The highly brilliant light source of the in-vacuum tapered undulator and the servomotor-driven channel-cut crystal compact monochromator resulted in satisfactory photon flux, energy resolution, energy range and data quality for *in situ* time-resolved and spatially resolved XAFS and XRD measurements. A time resolution of a few seconds was obtained by quick-scanning XAFS, and a spatial resolution of 1 µm was obtained by micro-beam mapping. The HAXPES now being installed will reveal the surface structure of electrode materials in conjunction with grazing-incidence XAFS and XRD. The dynamic electrochemical reactions inside LIBs can be investigated by developing advanced *in situ* techniques. Preliminary results revealed that BL28XU has the performance needed to serve as a total platform for analyzing battery reactions in detail and for supporting development of innovative rechargeable battery technologies.

## Figures and Tables

**Table 1:** Beamline details

Name	BL28XU
Source type	Tapered in-vacuum undulator
Mirrors	Rh and Pt stripe coated Si mirrors; M1: liquid-nitrogen-cooled, 1 m-long, flat; M2: water-cooled, 1 m-long, horizontal focusing; M3,M4: water-cooled, 700 mm-long, vertical focusing
Monochromator	Servo-motor-driven monochromator with 3 mm-gap, channel-cut Si (111) crystal
Energy range	5–30 keV
Beam size (uncollimated)	1 mm (V) × 2 mm (H)
Beam size at sample in each hutch focused by M3 and M4	0.1 mm (V) × 0.2 mm (H)
Beam size with KB mirror	1 µm
Flux at 8 keV (photons s^−1^)	1 × 10^14^, 1 × 10^11^ (collimated by KB mirror)
